# Reprogramming T-Cell Metabolism for Better Anti-Tumor Immunity

**DOI:** 10.3390/cells11193103

**Published:** 2022-10-01

**Authors:** Yu Ping, Chunyi Shen, Bo Huang, Yi Zhang

**Affiliations:** 1 Biotherapy Center, The First Affiliated Hospital of Zhengzhou University, Zhengzhou 450052, China; 2 Department of Immunology & National Key Laboratory of Medical Molecular Biology, Institute of Basic Medical Sciences, Chinese Academy of Medical Sciences (CAMS) & Peking Union Medical College, Beijing 100005, China

**Keywords:** T cell, tumor microenvironment, T cell metabolism, anti-tumor function

## Abstract

T cells play central roles in the anti-tumor immunity, whose activation and differentiation are profoundly regulated by intrinsic metabolic reprogramming. Emerging evidence has revealed that metabolic processes of T cells are generally altered by tumor cells or tumor released factors, leading to crippled anti-tumor immunity. Therefore, better understanding of T cell metabolic mechanism is crucial in developing the next generation of T cell-based anti-tumor immunotherapeutics. In this review, we discuss how metabolic pathways affect T cells to exert their anti-tumor effects and how to remodel the metabolic programs to improve T cell-mediated anti-tumor immune responses. We emphasize that glycolysis, carboxylic acid cycle, fatty acid oxidation, cholesterol metabolism, amino acid metabolism, and nucleotide metabolism work together to tune tumor-reactive T-cell activation and proliferation.

## 1. Introduction

Tumor immunotherapy is a significant breakthrough in cancer treatment. In 2018, James Allison and Tasuku Honjo won the Nobel Prize for Physiology or Medicine [1]. They have identified the function of the main immunosuppressive checkpoints (CTLA-4 and PD-1) in T cells to demonstrate T-cell dysfunction in cancers that lay the groundwork for tumor immunotherapy. At present, immune checkpoint inhibitors (e.g., PD-1 and CTLA-4) have been successfully applied and have shown a good clinical response [2]. Although immune checkpoint inhibitors have revolutionized cancer treatment, certain issues in clinical applications are yet to be solved.

T cells are key mediators of immune checkpoint inhibitor therapy [3,4,5,6]. It has long been known that T cells play pivotal roles in monitoring and killing tumor cells in cancers. Upon recognizing tumor antigens, T cells are activated and amplified to exert anti-tumor effects. The anti-tumor response of a T cell exhibits a state of exhaustion, such as high expression of PD-1 and CTLA-4, in cancers [7,8]. T-cell metabolism is closely associated with T-cell activation and exhaustion [9,10]. Understanding T-cell metabolism may help improve the T cell immune response to tumors. Glucose, fatty acids, and amino acids are the primary nutrients that produce energy for T cell immune response [11,12,13]. However, T-cell metabolism has changed due to the tumor microenvironment (TME), resulting in T cell exhaustion [14]. In this review, we describe the metabolic regulation involved in T-cell anti-tumor function in cancer. We highlight how metabolism modulates T-cell function in the TME and discuss strategies targeting abnormal metabolic processes for retrieving the anti-tumor responses of T cells.

## 2. Metabolic Characteristics of T Cells during Anti-Tumor Response

**Figure 1:**
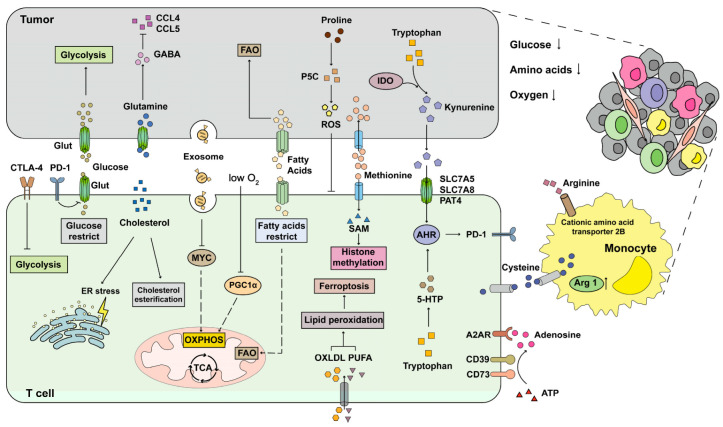
T-cell metabolism in the TME. The nutrients (glucose, fatty acids, and amino acids) in the TME are lacking due to tumor cells or immunosuppressive cells, leading to abnormal metabolism of T cells. Glucose and fatty acids are used in large quantities by tumor cells for proliferation, while the metabolism of glucose and fatty acids is restricted in the T cells that infiltrated the TME to reduced anti-tumor response. Immune checkpoints (PD-1 and CTLA-4) also inhibit glycolysis in the T cells that infiltrated the TME. ROS, kynurenine, PUFA, and OXLDL are enriched in TME to induce T cell ferroptosis and exhaustion. Hypoxia and exosome in TME inhibit PGC1α activation to damage T-cell anti-tumor function. Cysteine, arginine, and methionine are competitively ingested by tumor cells or monocytes to inhibit the T-cell anti-tumor response. ATP in TME is hydrolyzed by CD39 and CD73 to produce adenosine, which decreases the T-cell anti-tumor function through A2AR. Accumulated cholesterol in TME inhibits T-cell function through ER stress, and increased cholesterol esterification in T cells restricts the T-cell anti-tumor response. 5-HTP, 5-hydroxytryptophan—A2AR, A2A receptor—ER, endoplasmic reticulum—IDO, indoleamine 2,3-dioxygenase—OXLDL, oxidized low-density lipoproteins—PGC1α, PPAR-γ co-activator 1α—PUFA, poly-unsaturated fatty acids—ROS, reactive oxygen species—SAM, s-adenosylmethionine—TME, tumor microenvironment.

T cells become dysfunctional when they are influenced by the immunosuppressive environment in cancer. Abnormal cell metabolism is a major cause of T-cell dysfunction [15,16,17]. Numerous studies have shown that T-cell metabolism is aberrant along with a depleted anti-tumor response in cancer patients. Here, we mainly introduce the metabolic features of T cells in cancer (Figure 1).

### 2.1. Glucose Metabolism

Glucose is a necessary nutrient for T-cell growth and function. With prolonged T-cell activation, glucose uptake is enhanced by T-cell activation-associated signaling pathways, like CD28 or Akt, to maintain T-cell function [18]. However, to satisfy the replenishment of energy and substances for rapid proliferation, tumor cells take up large amounts of glucose for glycolysis, causing glucose depletion in TME [19], and glucose restriction in TME inhibits T-cell anti-tumor responses, causing tumor progression [20]. Glycolysis is the mainly metabolism for T-cell anti-tumor response. Glucose restriction reduces glycolysis to promote calcium reflux into the endoplasmic reticulum (ER) by causing the lack of phosphoenolpyruvate, resulting in diminished anti-tumor response [21]. Moreover, the high expression of inhibitory markers (e.g., PD-1 and CTLA-4) of the T cells that infiltrated the TME further restrict glucose uptake and utilization. For example, PD-1 and CTLA-4 signaling disturbs the transport and utilization of glucose in glycolytic metabolism [22]. Therefore, the enhanced glucose uptake of T cells or increased glucose in TME may improve T-cell anti-tumor function [13,23]. Additionally, due to the enhanced glycolysis of tumor cells, lactate is accumulated in TME. Lactate suppresses the proliferation and effector function of T cells [24], suggesting that improving T-cell function requires not only increasing glucose, but also removing lactate in the TME. Therefore, glucose deficiency is one of the main triggers for T-cell dysfunction in the TME.

### 2.2. Lipid Metabolism

Tumor cells exhibit abnormal lipid metabolism to affect T cell function in the TME [25]. It is also shown that tumor cells increase the uptake of fatty acids via the downregulation of prolyl hydroxylase-3 in high-fat diet tumor model to maintain the survival. This behavior inhibits T-cell infiltration and function [26]. In addition, some studies have reported that lipid is accumulated in the TME to play an immunosuppressive role [27,28]. T cells in tumor site highly expressed CD36, which promotes the uptake of poly-unsaturated fatty acids and oxidized low-density lipoproteins accumulated in the TME, inducing T cell ferroptosis and dysfunction [29,30].

Fatty acid metabolism is important for T cells in the TME. Enhanced fatty acid metabolism, such as fatty acid uptake, triglyceride synthesis, and fatty acid oxidation (FAO), mediated by peroxisome proliferator-activated receptor (PPAR)-α optimize the T-cell anti-tumor response to eliminate cancer cells [27]. Enhanced FAO also consolidates the T-cell anti-tumor response in the TME with low glucose and oxygen [27,31]. However, some studies have shown that FAO could prevent the anti-tumor response of T cells. PD-1 in exhausted T cell boosts FAO to restrain T cell effector function by activating STAT3 signaling, and leptin enriched in adipose tissues accelerates this process. Exogenous STAT3 signaling inhibitors and FAO inhibitors recover T cell effector functions to inhibit tumor growth [32]. T-cell senescence is a manifestation of T-cell dysfunction induced by both tumor and regulatory T-cells. RNA transcriptome data show that senescent T cells induced by regulatory T-cells exhibit increased FAO, fatty acid biosynthesis, and cholesterol biosynthesis [11]. Thus, FAO of T cell is a complex metabolism and shows distinct anti-tumor responses.

Memory T-cell is a small subset that is generated to maintain long-term protective immunity, which used fatty acid metabolism for survival [33]. Tissue-resident memory T (T_rm_) cell is a kind of memory T-cells that specifically reside in tissues and obtain energy for cell survival by relying on fatty acids. Tumor cells outcompete T_rm_ cells for the uptake of fatty acids to induce T_rm_ cell apoptosis, which is reversed by the PD-1 blockade antibody by increasing the fatty acid-binding protein (Fabp) 4/5 in T_rm_ cells and decreasing Fabp4/5 in tumor cells [34]. This finding indicates that fatty acid uptake by T_rm_ cells in the TME is impaired to restrict anti-tumor response.

In addition to fatty acids, cholesterol, which is the main component of the cell membrane, is an important lipid in T cells. Cholesterol is critical for anti-tumor response of T cells. Commanding cholesterol esterification by the decreasing enzyme ACAT1 maintains the cholesterol level in the plasma membrane to potentiate T-cell anti-tumor responses [35]. Tc9 cells, a small subset of secreted cytokine IL-9, have a stronger anti-tumor response than the classical effector T cells. The lower cholesterol content in Tc9 cells is maintained to eliminate tumor cells by decreasing cholesterol biosynthesis and increasing cholesterol efflux [36]. In addition, cholesterol is enriched in the TME to induce T-cell dysfunction. Cholesterol strengthens ER stress in T cells by activating ER stress sensor XBP1, to promote inhibitory immune checkpoint expression [37]. Therefore, understanding and reasonably improving the lipid metabolism in the TME can effectively improve the anti-tumor function of T cells.

### 2.3. Amino Acid Metabolism

Amino acids are the major substrates for protein biosynthesis. Under the long-term influence of the TME, certain amino acid metabolic pathways, including tryptophan, glutamine, arginine, methionine, and cystine, are unbalanced, which affect T-cell proliferation, survival, and cytotoxic function in the TME.

Tryptophan, an essential amino acid, is catalyzed to kynurenine via the heme enzyme indoleamine 2,3-dioxygenase (IDO). Tryptophan metabolism is thought to be an immunosuppressive factor that inhibits T cells and promotes tumor progression. It has been reported that IDO1 and catabolite kynurenine are highly expressed in tumors to accelerate T-cell dysfunction, which can be reversed by a decreased thymocyte selection-associated HMG box expression [38]. Ovarian cancer patients in The Cancer Genome Atlas (TCGA) with active tryptophan metabolism have poor clinical outcomes (overall survival and disease-free survival) [39]. Moreover, an IDO-mediated tryptophan metabolism not only affects the kynurenine metabolic pathway, but also influences other pathways such as purine, nicotinamide, and pyrimidine metabolism to lower T-cell function [39]. In addition to IDO, tryptophan 2,3-dioxygenase (TDO) is another enzyme involved in tryptophan catabolism. High TDO expression also impairs T-cell anti-tumor immunity and correlates with poor clinical prognosis [40]. 

Metabolite kynurenine inhibits T-cell infiltration and induces PD-1 expression by activating the aryl hydrocarbon receptor (AHR) to promote tumor progression [39]. The system L transporter SLC7A5 expressed in T cells promotes kynurenine uptake to constrain anti-tumor response [41]. A study of a chimeric antigen receptor (CAR) T cells showed that the high expression of IDO in the TME inhibits CAR-T cells proliferation and cytokine secretion via the metabolite kynurenine [42], which further demonstrate the inhibitory effect of tryptophan metabolism. Additionally, T cells in the TME can exacerbate the tryptophan metabolites production (kynurenine and 5-hydroxytryptophan). Cytokine IFN-γ secreted by T cell boosts the tumor-repopulating cells that exhibit high tumorigenicity and self-renews to release more kynurenine, which is transferred into T cells via SLC7A8 and PAT4 to increase PD-1 expression, accompanied by impaired anti-tumor immunity of T cells [43]. Furthermore, RNA transcriptome analysis of TCGA reveals that IL-2 expression is positively correlated with the T-cell exhaustion signature in different kinds of tumors. IL2 enhances tryptophan hydroxylase 1 expression, which catalyzes tryptophan as 5-hydroxytryptophan in T cells by activating the STAT5 signaling pathway to activate AHR [44]. Thus, manipulating tryptophan metabolism in TME can effectively increase T-cell anti-tumor response.

Glutamine is an important hub for biosynthetic precursors used for the anaplerosis of α-ketoglutarate, a critical intermediate in the tricarboxylic acid (TCA) cycle for maintaining the electron transport chain in activated T-cells and facilitating T-cell activation [45,46,47,48]. Hence, an intervention in the glutamine metabolism in tumor model enhances anti-tumor response of T cells in the TME. In the mouse tumor model, glutamine transporter inhibitor selectively inhibits glutamine uptake of tumor cell, and improves the uptake of glutamine and biosynthesis of glutathione for T-cell activation [49]. Moreover, glycolysis and the TCA cycle in T cells are replenished using glutamine antagonists [50]. In addition, glutamine in the TME is utilized by tumor cells expressed glutamate decarboxylase 1 to synthesize γ-aminobutyric acid. In addition, γ-aminobutyric acid further inhibits T-cell infiltration by suppressing chemokine expression (CCL4 and CCL5) [51,52]. Therefore, inhibition of glutamine metabolism alleviates malignancy behaviors of tumor cells [49,50]. 

Additionally, arginine consumption is important for T-cell activation. However, immunosuppressive cells (e.g., myeloid cells) and tumor cells in the TME plunder arginine via increased arginase 1 (Arg 1) to restrict T-cell anti-tumor function and survival [53,54,55]. It also has been reported that Arg 1 released in the TME directly inhibits T-cell receptor signaling transduction by downregulating the CD3ξ chain and high expression of cationic amino acid transporter 2B mediated arginine uptake in myeloid cells aggravates arginine deficiency in the TME [54]. Due to the low expression of ornithine transcarbamylase and argininosuccinate synthase, the viability of T cell further reduces in the arginine-deficient TME [56]. A recent study reported that, in an arginine-restricted TME, tumor cells and T cells exhibit distinct states based on Argininosuccinate synthetase 1 (ASS1) expression. ASS1, regulated by ATF4 and CEBPβ in tumor cells, is increased to enhance the synthesis of arginine from citrulline. However, ASS1 in T cells is still poorly expressed because of the tight chromosome that cannot bind to ATF4 and CEBPβ, resulting in impaired T-cell anti-tumor immunity [57]. Arginase 2 is another enzyme for arginine metabolism, and knockout of arginase 2 in T cells can increase anti-tumor function in nutrient-unbalanced TME [58]. These findings highlight the important link between arginine metabolism and T-cell function.

Other amino acids also exhibit abnormal metabolism in the TME. Exogenous cysteine is necessary for T cells because cysteine biosynthesis and cystine uptake are lacking in T cells. T cells uptake cysteine by transferring it from antigen-presenting cells via a neutral amino acid transporter, but antigen-presenting cells in the TME do not express neutral amino acid transporters and consume cysteine to restrain the cysteine uptake of T cells [59]. In addition, tumor cell can release 1-pyrroline-5-carboxylate, the downstream metabolite of proline dehydrogenase, to impair T-cell proliferation and cytokine secretion by increasing the number of reactive oxygen species (ROS) [60]. Intratumoral T-cell dysfunction induced by ROS is accelerated due to the lack of glutathione biosynthesis mediated by cystine metabolism [59,61,62]. Furthermore, tumor cells outcompete T cells for methionine uptake to maintain survival. Methionine deficiency alters histone methylation to inhibit T-cell survival and function [63]. Thus, an unbalanced amino acid metabolism in the TME is one of the main factors that induce T-cell dysfunction.

### 2.4. Nucleotide Metabolism

A nucleotide consists of three components: purine or pyrimidine, ribose or deoxyribose, and phosphoric acid. Purine and pyrimidine are also involved in the regulation of T-cell metabolism. For example, a one-carbon metabolism, such as purine and thymidine metabolism, is required for optimal T-cell proliferation [48,64,65]. Synthesis of pyrimidines mediated by dihydroorotate dehydrogenase contributes to T-cell expansion and effector molecule expression [66]. Meanwhile, adenosine triphosphate (ATP) released by T cells act on the P2X4 expressed on T cells to promote migration to antigen-presenting cells in the process of T-cell antigen recognition [67]. Furthermore, extracellular ATP is benefit for supporting the generation of long-lived CD8^+^ memory T-cells by binding purinergic receptor P2RX7 [68,69,70,71]. However, the nucleotide metabolism in the TME is abnormal. ATP in the TME is rapidly catalyzed by ecto-5′-nucleotidases (CD39 and CD73). T cells in the TME highly express CD39 and CD73, which accelerate the hydrolysis of ATP to adenosine [72,73]. Adenosine is the primary immunosuppressive metabolite in the TME that promotes tumor progression [74]. It is the adenosine receptor, the A2A receptor (A2AR), which is highly expressed in T cells in the blood and tumor tissues of cancer patients. Adenosine in the TME stimulates protein kinase A and the mTOR signaling pathway to inhibit T-cell cytokine expression by binding to A2AR [75]. Additionally, damaged cells can release purine nucleotides to affect T-cell proliferation and cytokines production [76,77]. Taken together, these studies show that T-cell anti-tumor immunity is modulated by nucleotide metabolites.

### 2.5. Mitochondrial Metabolism

The mitochondria are indispensable organelles in the TCA cycle and oxidative phosphorylation (OXPHOS) for energy supplement in T cells [33,78,79]. Memory T-cells have fused mitochondria, with high mitochondrial mass and tight cristae, to maintain long-time survival and immune response [33,79]. However, the mitochondrial morphology is aberrant with ROS accumulation in the TME, suggesting that the T-cell anti-tumor immunity is strictly astricted [80,81]. In addition, due to the tumor antigen persistent stimulation in the TME, mitochondrial function in T cell is impaired, inhibiting the electron transport chain [82]. The mitochondrial number of T cells in tumor tissues are lower and the cristae of mitochondria are shorter. Abnormal mitochondria induce T-cell dysfunction, which can be enhanced by the PD-1 signaling pathway [83]. PPAR-γ co-activator 1α (PGC1α) is a critical hub for mitochondrial biogenesis, OXPHOS, and ATP production. Nonetheless, T cells infiltrating tumors exhibit low expression of PGC1α, leading to depleted metabolism and anti-tumor response [84]. It has been reported that PGC1α expression in the T cells infiltrated the tumor site are repressed by hypoxia, which is one of the main features of the TME induced by the rapid proliferation and metabolism of tumor cells. Hypoxia also promotes ROS production in the mitochondria and induces T-cell dysfunction [85]. Furthermore, in the hypoxia TME, tumor cells secrete exosomes containing miRNA24 to inhibit the MYC-mediated signaling pathway that suppresses OXPHOS activity to induce T-cell dysfunction [86]. Importantly, OXPHOS activity can be further impaired by NADH, the carrier and electron donor for biological hydrogen in the electron transport chain. The NAD^+^ levels in intratumoral T-cells mediated by nicotinamide phosphoribosyltransferase are decreased, resulting in impaired OXPHOS [87]. Therefore, abnormal mitochondrial metabolism is a determinant of T-cell dysfunction.

## 3. Targeting Metabolism to Improve T Cell Mediated Anti-Tumor Efficiency

**Figure 2:**
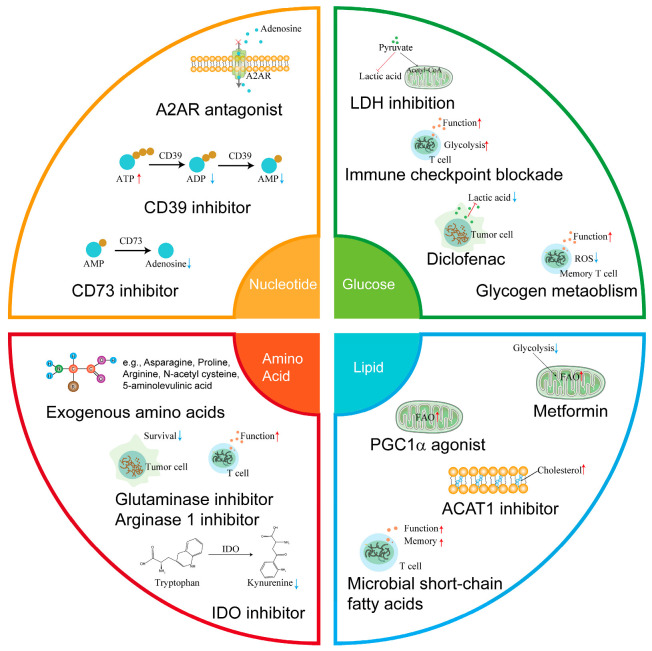
Metabolic intervention strategy for improving the anti-tumor response of T cells. The interventions of glucose, lipid, amino acid, and nucleotide metabolism have been used to enhance T-cell anti-tumor responses. A2AR, A2A receptor—IDO, indoleamine 2,3-dioxygenase—LDH, Lactate dehydrogenase—PGC1α, PPAR-γ co-activator 1α.

Aberrant metabolism of the TME limits T-cell anti-tumor responses. Many studies have identified that manipulating metabolism in the TME reinvigorate anti-tumor immunity of intratumoral T-cells (Figure 2).

### 3.1. Targeting Glucose Metabolism in the TME

Correction of glucose metabolism in the TME recovers the anti-tumor response of T cells. Immune checkpoint blockade has been proved to rescue T-cell glycolysis for improving the anti-tumor response of the T cells that infiltrated the TME [19]. However, the enhanced tumor oxidative metabolism leads to the poor responsiveness of immune checkpoint blockade to promote tumor progression [88]. These findings suggest that immune checkpoint blockade is not an effective means for enhancing intratumoral T-cell glycolysis. Moreover, restricting T-cell glycolysis by inhibiting glycolysis associated enzyme phosphoglycerate mutase-1 or using glucose analog 2-DG enhance T-cell anti-tumor response by promoting the formation of memory T-cells [89]. Based on these data, to enhance T-cell anti-tumor response by targeting glucose metabolism, the T-cell subset and tumor metabolism should be considered.

The enhanced glycolysis of tumor cell induces accumulated lactate in the TME to inhibit T-cell anti-tumor response [24,90]. Lactate dehydrogenase (LDH) catalyzes pyruvate to lactate. Inhibiting LDH can decrease lactate production and promote pyruvate entry into the TCA cycle to enhance the T-cell anti-tumor response [91]. Diclofenac, a non-steroidal anti-inflammatory drug, decreases the lactate release of tumor cells by inhibiting lactate transporters monocarboxylate transporter to improve T-cell anti-tumor function [92]. These findings support that the anti-tumor function of T cells can be enhanced by decreasing lactate in the TME.

ROS are accumulated in the TME to impair the anti-tumor function of T cells. It has been reported that glycogen metabolism is a key metabolic pathway that eliminates ROS and maintains the immune response of memory T-cells [93,94]. The CD8^+^ memory T-cells exhibit increased intracellular glycogen via gluconeogenesis mediated by glycogen phosphorylase and phosphoenolpyruvate carboxykinase 1 (Pck1) [95,96], and glycogen contributes to the production of glutathione to neutralize ROS through PPP [95]. Moreover, mitochondrial acetyl coenzyme A indirectly increases Pck1 expression via ketogenesis-derived β-hydroxybutyrate to promote gluconeogenesis in memory T-cells, thereby maintaining the immune response of memory T-cells [97]. These results highlight the indispensable role of glycogen metabolism in memory T-cells and enhanced T-cell glycogen metabolism may support memory T-cells’ survival and anti-tumor response in the TME.

### 3.2. Targeting Fatty Acid Metabolism

Targeting fatty acid metabolism may return the T cells’ anti-tumor function in the TME. A tumor vaccine vector containing an encapsulating tumor antigen, metformin and hollow gold nanospheres is constructed for cancer treatment, and metformin in the tumor vaccine has been proven to improve cancer therapy and enhances memory T-cell differentiation by promoting glycolysis into FAO in mouse tumor model [98], indicating that lipid metabolism intervention improves T-cell anti-tumor function. PPAR signaling pathway plays a vital role for increasing FAO in T cells. In mouse tumor model, bezafibrate, a PPAR signaling agonist, may maintain T-cell numbers by enhancing FAO and improves the therapeutic effect of PD-1 blockade antibody [31]. Thus, the enhanced FAO can optimize anti-tumor response of T cells in the TME. In addition, targeting cholesterol metabolism has benefit for T-cell function. Avasimibe, an inhibitor of the cholesterol esterification enzyme ACAT1, increases cholesterol levels in the cell membrane, facilitating the formation of immune synapses and signal transduction, resulting in superior anti-tumor efficacy in different mouse models [99,100].

Microbial short-chain fatty acids are essential for the T cell’s anti-tumor function. Supplements of microbial short-chain fatty acids inhibit tumor growth by enhancing the T-cell’s effector molecule expression [101]. Moreover, microbiota-derived fatty acids are critical for the recall response and differentiation of memory T-cells, which affect the FAO and OXPHOS in memory T-cells [102]. Therefore, microbial short-chain fatty acids may facilitate anti-tumor response of T cells via enhancing an effector molecule expression or memory formation.

### 3.3. Targeting Amino Acid Metabolism

Exogenous supplementation of amino acid shows a good anti-tumor effect by activating mitochondrial metabolism of T cells, and protects T cells from exhaustion and enhancing T-cell proliferation and activation, such as 5-aminolevulinic acid, N-acetyl cysteine, asparagine, proline, and arginine [103,104,105,106,107]. At present, some drugs targeting amino acid metabolism have been developed for anti-tumor treatment. Glutaminase inhibitor CB-839 has been used in preclinical experiments and clinical trials [108,109,110,111]. Experiments in mouse tumor models have shown that CB-839 treatment improves the anti-tumor efficiency of tumor antigen-specific T cells [108]. A phase I clinical trial of PI3K3CA-mutant colorectal cancers showed that the combination of CB-839 and capecitabine exhibits a good therapeutic effect [109]. The arginase 1 inhibitor INCB001158 promotes T-cell infiltration and inflammatory cytokines production in the TME in the mouse tumor models [112]. It indicates that the regulation of arginase metabolism contributes to restore the T cell’s anti-tumor function. Furthermore, the clinical trials of pegylated arginine deiminase (ADI-PEG 20), which can lower arginine in cancer patients, have been performed [113,114,115,116,117]. In the clinical trial of ADI-PEG 20 plus pembrolizumab, T cell infiltration in the TME is increased by ADI-PEG 20 treatment in advanced solid cancers with low PD-L1 expression of TME [113].

IDO has a wide range of inhibitory effects on various tumors. In mouse tumor models, inhibiting IDO can promote T-cell infiltration and increase the cytokines production [118,119]. The IDO1 inhibitor epacadostat has been reported to improve the anti-tumor effect of CAR-T cells in esophageal squamous cell carcinoma [120]. The clinical trial of epacadostat plus immune checkpoint blockade antibody have been studied the furthest [121]. Epacadostat in combination with ipilimumab exhibits enhanced clinical response in the phase I/II clinical trial of patients with unresectable or metastatic melanoma [122]. However, in a phase III clinical trial, compared with the placebo plus pembrolizumab group, epacadostat plus pembrolizumab do not show good therapeutic effects in patients with unresectable or metastatic melanoma [123]. Moreover, although epacadostat or PI3Kδ inhibitor combined with JAK1 inhibitor induce several changes in the TME, there are limited effects on the anti-tumor response in advanced solid tumors in phase I clinical trials [124]. Thus, IDO-mediated kynurenine metabolism needs to be further investigated for clinical applications.

### 3.4. Targeting Adenosine Metabolism

Adenosine is markedly enriched in the TME to restrain T-cell anti-tumor response via A2AR [74]. CD39 and CD73 expression in the TME catalyze ATP to AMP and AMP to adenosine, respectively [125]. Thus, targeting adenosine metabolism facilitates the restoration of T-cell anti-tumor function, such as CD39 inhibitors, CD73 inhibitors and A2AR antagonist. A number of preclinical studies have revealed that anti-tumor response of T cell can be restored by targeting CD39, CD73 or A2AR [126,127,128,129,130]. For example, CD39 and CD73 inhibitors (IPH5201 and IPH5301) decrease adenosine levels and maintain the ATP content to enhance T-cell activation [131]. Anti-CD39 antibody or CD39 specific antisense oligonucleotides improve intratumoral T-cell function to control tumor progression [130,132]. CPI-444, an A2AR antagonist, combined with immune checkpoint blockade antibodies (PD-L1 and CTLA-4), significantly reduces the rate of tumor growth by improving T-cell anti-tumor immune responses [133].

At present, several clinical trials of targeting adenosine metabolism have been performed. The combination of durvalumeb and anti-CD73 antibody oleclumab improve objective response rate and progression-free survival in phase II clinical trial of unresectable stage III non-small-cell lung cancer [134]. In phase I clinical trial of patients with renal cell cancer, durable clinical benefits and increased T-cell infiltration into the TME were observed in combination with atezolizumab and A2A2 antagonist ciforadenant (CPI-444) [135]. Hence, interfering with the adenosine metabolism, including the CD73 inhibitors or A2AR antagonist, may enhance anti-tumor immunity in clinical settings.

## 4. Concluding Remarks

Cell metabolism plays an irreplaceable role in T-cell activation. However, the TME shows depleted nutrients and enriched immunosuppressive metabolites to restrict the T-cell anti-tumor response. Understanding and manipulating T-cell metabolism in the TME is beneficial to improve T-cell anti-tumor function. Evidently, metabolic interventions have been reported to inhibit tumor progression by enhancing T-cell proliferation, survival, and function in preclinical experiments and clinical trials. Therefore, metabolic interventions not only protect T cells from dysfunction, but also provide therapeutic strategies for cancer. Further studies on metabolic interventions are needed to explore the anti-tumor effectors of T cells.

## Data Availability

Not applicable.
